# Supplementary material for: Uniform dynamics of cohesin-mediated loop extrusion in living human cells
Source: Nat Genet. 2025 Nov 14;57(12):3152–64. doi: 10.1038/s41588-025-02406-9 (PMC12695666; doi:10.1038/s41588-025-02406-9)
Supplement: Supplementary file 1 — Supplementary Methods and Supplementary Fig. 1. [file 41588_2025_2406_MOESM1_ESM.pdf]

nature genetics

Supplementary information https://doi.org/10.1038/s41588-025-02406-9

# Uniform dynamics of cohesin-mediated loop extrusion in living human cells

In the format provided by the authors and unedited

# Table of contents

**METHODS** ..... **2**

COMPUTATIONAL METHODS ..... 2

*Genomic data analysis* ..... *2*

*Choice of genomic loci for labeling of TAD anchors* ..... *2*

*Image analysis* ..... *3*

Image shift correction ..... 3

Eliminating replicated spots ..... 4

Detecting and tracking fluorescent spots ..... 5

Correcting for chromatic aberrations ..... 7

Refining localizations and measuring localization precision ..... 8

Quality filtering of time series ..... 9

Scoring of localization precision ..... 9

Visualization of images ..... 10

*Quantification of distance time series* ..... *10*

2-point Mean-Squared Displacement ..... 10

Proximal state segmentation ..... 11

Computing fraction, frequency and lifetime of proximal states ..... 13

Estimation of loop state fractions ..... 13

Model-free estimation of the maximal fraction of open states ..... 15

Estimation of closing rate ..... 16

*3D polymer simulations of loop extrusion* ..... *18*

Molecular dynamics simulations ..... 18

Loop extrusion modeling ..... 19

Modeling CTCF residence on chromatin ..... 20

Comparison of simulations to experiments ..... 21

Characterization of proximal and closed states ..... 23

**SUPPLEMENTARY TABLES** ..... **25**

**SUPPLEMENTARY VIDEOS** ..... **26**

**SUPPLEMENTARY FIGURE 1** ..... **27**

**REFERENCES** ..... **28**

# Methods

## Computational methods

### Genomic data analysis

The hg19 genome was used for all genomic analyses. HCT116 genomic data were retrieved from *Rao et al*[1]. Loops and TADs were called using HiCCUPS and Arrowhead, respectively, from Juicer 1.19.02[2]. The following flags were used for HiCCUPS: -r5000, 0000 -k KR -f 0.1 -p 4,2 -I 7,5 -t 0.02,1.5,1.75,2 -d 20000, 20000; and for Arrowhead: -m 2000 -r 5000 -k KR --threads 10.

For ChIP-Seq data of CTCF, SMC1 and RAD21, we used publicly available data from *Rao et al*[1]. Raw reads were quality-checked using FastQC[3]. Reads from different replicates were first mapped independently using Bowtie2 v2.2.6.2[4] with default parameters, and the correlation between replicates was computed using wigCorrelate[5]. Replicates with correlations larger than 0.9 were pooled together and mapped again. We removed blacklisted regions[6] and called peaks using default parameters of MACS2 v2.1[7]. For ChIP-Seq of histone marks, we used the flag 'broad'. CTCF motifs were identified genome-wide using FIMO v5.3.0[8] with the flags -max-stored-scores 50000000 and -thresh 0.001 and the Jaspar motif MA0139.1. We then mapped CTCF sites identified with a P-value < $1x10^{-5}$ onto CTCF ChIP-Seq peaks.

ChIP-Seq peaks of CTCF, SMC1 and RAD21 were intersected with regions of 20 kb centered around TAD anchors using pgltools v2.7.1[9].

A and B compartments were identified using 'eigenvector' from Juicer with the flags KR BP 100000. A compartments were defined as genomic regions with high H3K27ac, Pro-Seq, H3K36me3, H3K4me3 and H3K4me1 signals and B compartments as the opposite.

### Choice of genomic loci for labeling of TAD anchors

Using Hi-C, ChIP-Seq and PRO-seq data from Rao *et al*[1], we filtered loops and TADs based on the following criteria: (i) size comprised between 300 kb and 1.5 Mb, (ii) at least

one peak of SMC1, RAD21 and CTCF at both anchors, (iii) at least one pair of convergent CTCF sites at the anchors, (iv) no gene at anchors and (v) low gene expression within the domain (<1.5 reads per kilobase million, RPKM). These criteria were defined to minimize possible cohesin-independent chromatin interactions that might obscure cohesin-dependent extrusion dynamics. From this subset, to further ensure that selected domains were cohesin-dependent, we removed domains exhibiting enhancers at their anchors (as identified in the GeneHancer double elite set[10]). After filtering, we obtained a list of 96 loops and 32 TADs, from which we manually removed highly nested domains and domains containing alignment artifacts in Hi-C maps. We then selected loops and TADs (Extended Data Fig. 1d,e) for fluorescent tagging. This selection was based on the following criteria: (i) strong TAD or loop insulation scores, (ii) genomic regions exhibiting highly ranked sgRNAs based on ChopChop[11] scoring to facilitate genome editing and (iii) sgRNAs sites at less than 5 kb from CTCF anchors to ensure the most accurate readout of anchor-anchor distances, as previously assessed by polymer simulations[12,13].

For the Adjacent locus, we inserted the TetOx96 repeats 6 kb away (mid-array distance) from the CuOx150 repeat array used to label the 3' anchor of the T1 TAD (Extended Data Fig. 1a). For the Half TAD locus, we inserted the TetOx96 repeats on the 5' side of the 5' anchor of the L1 TAD in a cell line that already contained the CuOx150 repeat array at the 3' CTCF border. No CTCF site is present within 100 kb and no convergent CTCF site relative to the 3' anchor was identified within 117 kb of the TetOx96 repeat array insertion site (Extended Data Fig. 1a).

## Image analysis

### Image shift correction

Due to misalignment of the motorized microscope stage device, small shifts can appear between consecutive 3D image stacks when imaging multiple fields of views. While axial shifts are avoided thanks to the perfect focus system, lateral shifts can reach a few hundred nanometers, negatively affecting anchor tracking. To correct these lateral shifts, we first computed 2D cross-correlations between the 2D maximum intensity projections

of the z-stack at each timepoint and the first timepoint image (the green and far-red channels were summed), and then used the estimated displacement to shift both the green and the far-red channel images with pixelic precision and without pixel interpolation. Please note that this correction does not affect the calculation of distances between the fluorescently labeled anchors but helps alleviate tracking problems.

### Eliminating replicated spots

We manually eliminated cells that started their S phase and dividing cells based on the observation of replicated chromatin loci as follows. First, we eliminated cells in which maximum intensity projections showed two distinct spots, indicating replicated loci, by simple visual inspection. However, two replicated loci may not always be resolvable if their distance is below the diffraction-limited resolution. To identify these more complex cases, we reasoned that a round spot likely corresponds to a single chromatin locus whereas an elongated spot likely corresponds to two overlapping spots from two replicated chromatids. We therefore computed the elongation of the detected spots and removed cells with a large spot elongation (Extended Data Fig. 2e), as follows. Spot elongation was measured in two steps. First, a 3D Laplacian of Gaussian filter was applied to the 3D image stacks, and spots were detected by searching for local maxima above a given threshold. Second, we fitted a 3D second order polynomial function (paraboloid) in a region of 9x9x9 voxels around each local maximum. We selected 8 points at 45° intervals around the vertex of the paraboloid such that their amplitude is 0.9 times the peak intensity at the vertex, and we estimated the covariance among these 8 positions within the XY, XZ and YZ planes. For each of these 3 planes, we computed the ratio between the largest and smallest eigenvalues of the covariance matrix minus 1 and defined the largest score of the three ratios as the elongation score. An elongation score of 0 corresponds to an isotropic spot, whereas a large score corresponds to an elongated spot. We computed this score for each spot in the far-red channel and for each time lapse, and created a single 2D image using a projection of these scores along the time-axis and the z-axis (Extended Data Fig. 2e). This image served as a guide for the above-mentioned

visual elimination of replicated spots. We verified that our method to eliminate replicated spots was conservative, as the fraction of retained cells in imaging data was slightly lower than the fraction of G1 cells measured by FACS (Extended Data Fig. 2f,g). We note that we cannot eliminate cells that started the S phase but where the labelled locus was not yet replicated. Noteworthy, we consistently observed a lower fraction of cells in G1 in auxin-treated cells as compared to untreated cells, as expected from cell cycle arrest due to RAD21 depletion[14].

**Detecting and tracking fluorescent spots**

To detect and track fluorescent spots, we processed the 3D image time series using Fiji plugins as follows. First, we manually defined rectangular Regions Of Interest (ROIs) around each pair of spots corresponding to cells in G1 or early S phase. A single ROI was used for each pair of spots, *i.e.* each ROI contained all positions explored by the locus during the entire time lapse acquisition.

Detection and tracking of fluorescent spots were performed separately for each color channel using TrackMate[15] (Extended Data Fig. 2a). To improve the detection of spots in images of variable Signal-to-Noise Ratio (SNR) relative to TrackMate's default Laplacian of Gaussian detector, we implemented a spot detector based on the determinant of the local Hessian matrix. The resulting Hessian detector is more robust to spurious local intensity maxima that tend to occur at the edge of bright objects[16], such as nuclei. We used a different filter size in Z and XY, to account for the axial elongation of the PSF[17]. In order to facilitate removal of spurious detections, the 'quality' score computed by TrackMate was normalized within the ROI. Detections with quality scores below 0.7 and 0.8 for Halo and GFP, respectively, were rejected. We then used a localization algorithm based on parabolic interpolation[18] to refine the spot position with sub-pixel precision.

Next, we connected detected spots over time to generate trajectories. This was done using the simple Linear Assignment Problem (LAP) tracker[19] in TrackMate with the following parameters: maximum linking distance = 0.9 $\mu$m, maximum gap-closing distance = 1.4 $\mu$m and maximum gap-closing frame gap = 12 frames. Spurious detections

due to noise typically generated short trajectories. To remove them, we set the minimum number of detections per trajectory to ~20 for the GFP channel and ~40 for the Halo channel. The resulting trajectories possibly contained large gaps, *i.e.* several consecutive timepoints with detections below the above-mentioned quality score thresholds. To address this, we implemented a gap-filling step in TrackMate, where trajectories with gaps were automatically revisited and corrected as follows. For each gap, we first used linear interpolation between the 3D spot positions immediately before and after the gap. Second, we performed another detection with the Hessian detector within the gap interval, but restricted to a distance of at most 0.5 $\mu$m from these interpolated positions. We then added the detection (if any) with the highest quality score above the quality threshold to the trajectory and updated the interpolated positions in the remaining gaps. This led to a partial closure of the trajectory gaps, although smaller gaps typically remained when no detection of sufficient quality score was found within the search space. The detection and tracking parameters were optimized automatically with the TrackMate-Helper plugin[20] using manually annotated images as ground truths. Specifically, parameters were optimized to maximize the 'matching score against ground truth trajectories, penalizing spurious tracks', a metric previously defined in the single particle tracking challenge[21]. After tracking each channel and before pairing of the two channels, the trajectory coordinates were corrected for chromatic aberrations (see section 'Correcting for chromatic aberrations' below and Extended Data Fig. 2a,b).

Subsequently, the 3D trajectories determined independently in the green and far-red channels were paired together using a custom-written Fiji plugin "Pair TrackMate files", as follows. For each trajectory in the green channel, we considered all far-red channel trajectories that overlapped in time and counted the number of timepoints for which the detected green and far-red spots were within 2.5 $\mu$m of each other. The trajectory pair with the largest number of such timepoints was retained and both trajectories were removed from the list of candidate trajectories. This procedure was repeated until either the list of green channel or far-red channel trajectories was empty. The 3D coordinates of each spot in the paired trajectories were then refined by maximum likelihood estimation (see section 'Refining localizations and measuring localization precision' below).

All tools described here are publicly available in Fiji[22], either in the TrackMate plugin, or in the two extensions "TrackMate-Helper" and "TrackMate-Pairing", available by subscribing to the Fiji update sites of the same name.

### Correcting for chromatic aberrations

Correcting chromatic aberrations is crucial to precisely compute the 3D distance between the far-red and green loci. To estimate chromatic aberrations in 3D in the exact same plate and medium used for imaging TAD anchors, we acquired 3D reference images of actin in WT HCT116 cells in the green and far-red channels using CellMask green and Deep Red actin stains (ThermoFisher A57245 and A57243, respectively; Extended Data Fig. 2a). At least ten fields of view of actin z-stacks were acquired every second day of imaging. Chromatic shifts were then measured with Chromagnon v0.94[23] using the 3D actin images averaged over all fields of view as references and the option 'Local align' set to 'None'. The estimated 3D XYZ translations, 3D magnifications and 2D lateral rotations were used to correct spot localizations (Extended Data Fig. 2b).
In order to quantify this actin-based chromatic aberration correction (and for this purpose only), we acquired timelapse images of fluorescent beads (Tetraspeck 0.1 $\mu$m, T7279) in the green and far-red channels using the same imaging parameters as for live-cell imaging of chromatin loci. Beads were positioned on the bottom of the imaging plate in PBS. Beads were then detected and localized using the same methods as for the chromatin loci. Next, we corrected bead localizations for chromatic aberrations as described above and computed the 3D distance between the two color channels for each fluorescent bead (Extended Data Fig. 2a,b). We found that we could accurately correct for chromatic aberration since the median 3D distance decreased from 265 nm before correction to 50 nm after correction (Extended Data Fig. 2b). This highlights the importance of correcting chromatic aberrations to study sub-micrometric distances between chromatin loci imaged in different color channels.

## Refining localizations and measuring localization precision

All paired green and far-red channel localizations obtained using the above methods, including interpolated missing positions, were refined using maximum likelihood estimation (MLE), which is known to be optimal for precise localization when the PSF and noise models are known[24]. Because our MLE algorithm assumes Poisson noise, but our EMCCD cameras do not provide photon counts, we first converted the pixel values into photon counts. For this purpose, we acquired 100 images of the same field of view of fixed cells, measured the mean and variance of each pixel, and determined the affine function which, when applied to the pixel intensities, yields a mean approximately equal to the variance at each pixel. All spots were localized by MLE using an anisotropic 3D Gaussian PSF with different lateral and axial standard deviations, $\sigma_{\mathrm{x,y}}$ and $\sigma_{\mathrm{z}}$, which was calibrated as follows. The iterative MLE algorithm was initialized using the spot positions identified by TrackMate or by interpolation as described above, and was performed in a 3D region of 7x7x7 voxels centered on these positions (Extended Data Fig. 2a). To determine $\sigma_{\mathrm{x,y}}$ and $\sigma_{\mathrm{z}}$, we first performed MLE by fitting the 3 spot coordinates, the amplitude, and a constant background together with the lateral and axial standard deviations. In a second step, we fixed $\sigma_{\mathrm{x,y}}$ and $\sigma_{\mathrm{z}}$ to the medians of the estimated values on each cell line (pooling untreated and auxin-treated cells together) and performed MLE again to estimate the 3 coordinates, the amplitude and the background. Furthermore, for each coordinate $v \in \{x, y, z\}$, we computed the Cramér-Rao bound[24] $e_{c,v}$, providing us a lower bound $e_{c,v} = \sqrt{\langle(\hat{v} - v)^2\rangle}$ of the localization precision in nanometer for each fluorescent spot and each channel $c \in \{r, g\}$ at each time point. The localization precision of a coordinate $v$ measures the standard deviation of the localization error, *i.e.* $\langle(\hat{v} - \langle\hat{v}\rangle)^2\rangle^{1/2}$, where $\hat{v}$ denotes the estimation of $v$ and $\langle\cdot\rangle$ denotes statistical averaging. The 3D localization precision was then computed as $e_c = \sqrt{e_{c,x}^2 + e_{c,y}^2 + e_{c,z}^2}$ and the precision on distances measured between the far-red and green loci was computed as $e_d = \sqrt{e_r^2 + e_g^2}$ (Extended Data Fig. 2d). Localization precision was used as a measure of the uncertainty associated with each localization and included in all downstream analyses.

## Quality filtering of time series

Before analysis, time series were quality filtered to minimize localization and tracking errors (Extended Data Fig. 2a,c). First, we removed localizations with poor precision in each channel ($e_c$ > 250 nm), pairs of localizations with poor distance precision ($e_d$ > 250 nm), and localizations at the edge of the volume used for Gaussian fitting (< 1 pixel from volume edge). To filter out stepwise tracking errors, we computed the frame-to-frame displacement of each fluorescent spot and the frame-to-frame change in 3D distance between the two spots. Timepoints that simultaneously exhibited a z-score > 1.75 for the change in distance and at least one of the two displacements were considered as tracking errors and corresponding localizations were removed. Moreover, we trimmed the end of time series such that they did not contain gaps of more than 10 consecutive missing localization pairs. After removal of single timepoints, we then filtered out short and low-quality time series by removing: (i) time series with less than 20 timepoints, (ii) time series with a median distance precision $e_d$ > 150 nm, (iii) time series with more than 30% of missing time points. Residual trajectory pairing errors were then filtered out by removing time series with a median distance between the far-red and green spots exceeding 1.5 $\mu$m (except for the adjacent cell line where we used a threshold of 1 $\mu$m).
Finally, remaining gaps in time series were filled by interpolating the distance between the time points immediately before and after the gaps. These interpolated distances represented on average 8.6% of timepoints across all conditions (6-13% is the minimum to maximum among all cell lines and treatments).

## Scoring of localization precision

We assigned a weight to each computed anchor-anchor distance based on the localization precision $e_d$ as follows:

$$w(e_d) = 1 - \frac{1}{2}\left[1 + \mathrm{erf}\left(\frac{e_d - (\mu_{e_d} + \sigma_{e_d})}{\sqrt{2}\sigma_{e_d}}\right)\right] \in [0,1],$$

where $\mu_{e_d}$ and $\sigma_{e_d}$ stand for the mean and standard deviation, respectively, of $e_d$ computed across all cell lines and treatments and $\mathrm{erf}$ is the error function. Better localization precisions (lower $e_d$) of far-red and green spots lead to higher weights, and distances measured with better precision thus contribute more to subsequent analyses than less precise distance measurements.

### Visualization of images

Solely for visualization purposes (Fig. 1d,e, Extended Data Fig. 2a,e and 3a, Supplementary Video 1), time lapse images were corrected for photobleaching by the exponential fitting function of the ImageJ 'Bleach Correction' plugin.

## Quantification of distance time series

### 2-point Mean-Squared Displacement

The 2-point MSD at a time lag $k\delta\mathrm{t}$ (where $k$ is an integer and $\delta\mathrm{t} = 30s$ is the sampling time interval) for a single trajectory with $m$ time points was computed as:

$$2\mathrm{pMSD}(k\delta\mathrm{t}) = \frac{1}{m-k}\sum_{i=1}^{m-k}\left(\boldsymbol{d}\big((i+k)\delta\mathrm{t}\big) - \boldsymbol{d}(i\delta\mathrm{t})\right)^2$$

where $\boldsymbol{d}(t) = \boldsymbol{r}_{\mathrm{red}}(t) - \boldsymbol{r}_{\mathrm{green}}(t)$ is the 3D vector linking the positions of the far-red and green spots at time $t$ and $m-k$ is the number of overlapping time intervals of length $k\delta\mathrm{t}$ contained within the time series. For a set of $n > 1$ trajectories, the average 2-point MSD is:

$$2\mathrm{pMSD}(k\delta\mathrm{t}) = \left(\sum_{j=1}^{n} m_j - k\right)^{-1}\sum_{j=1}^{n}\sum_{i=1}^{m_j-k}\left(\boldsymbol{d}_j\big((i+k)\delta\mathrm{t}\big) - \boldsymbol{d}_j(i\delta\mathrm{t})\right)^2$$

where $\boldsymbol{d}_j(t)$ is the 3D vector linking the positions of the far-red and green spots at time $t$ in trajectory $j \in [1, n]$ and $m_j$ is the number of timepoints in trajectory $j$.

## Proximal state segmentation

To segment distance time series into intervals of proximal states, we used a simple, model-free approach involving a spatial and a temporal threshold, which we previously described and validated on polymer simulations[12]. Proximal state intervals were identified as time intervals during which the 3D anchor-anchor distances remain below the spatial threshold for a duration at least equal to the temporal threshold.

The spatial threshold was defined using a theoretical polymer model of the closed state distance distribution and was set to the 95$^{th}$ percentile of this distribution. We built this theoretical closed state distribution by simulating $N=10^7$ pairs of 3D spot positions and sequentially adding three sources of errors as follows. First, we simulated the 3D positions of the two anchors assuming that they are separated by a distance of 40 nm, corresponding to the cohesin ring size[25,26] and consistent with the expected nontopological entrapment of DNA within its ring[27,28]. Second, to account for the chromatin linker between TAD anchors and fluorescent reporters, we shifted each position by a random displacement following a normal distribution determined based on the known genomic distance between the anchor and the reporter, and assuming a Kuhn length of 40 nm (in agreement with experimental estimations of 32-268 nm[29–33]) and a chromatin compaction of 44 bp/nm (in agreement with estimations of 18-66 bp/nm[29,31,32]). Third, to account for random localization errors, we added normally distributed 3D random displacements with lateral and axial standard deviations randomly drawn from the n=12,269-93,431 localization precisions estimated by Cramér-Rao bounds on each cell line. Thus, our theoretical distribution of distances in the closed state takes into account the observed small differences of localization errors (Extended Data Fig. 2d and Supplementary Table 1) and reporter-anchor separations (Supplementary Table 2) between the different cell lines. This model was also used in Extended Data Fig. 4c to predict the influence of persistence length on distances in the Adjacent locus. With the assumed 95$^{th}$ percentile, these distributions resulted in spatial thresholds of 0.199, 0.218, 0.236, 0.220 and 0.249 $\mu$m for L1, L2, T1, Half TAD and the Adjacent locus, respectively (Supplementary Table 2). For the stringent dataset (Extended Data Fig. 6e,g,h), the same

percentile resulted in slightly lower spatial thresholds of 0.191, 0.202, 0.217, 0.196 and 0.216 $\mu$m for L1, L2, T1, Half TAD and the Adjacent locus, respectively, because of the better localization precisions in this dataset (Supplementary Table 2).

The temporal threshold was defined as the minimal time interval during which 3D anchor-anchor distances remain smaller than the spatial threshold, which allowed to minimize false identification of closed states due to stochastic motion of chromatin alone[12]. We set the temporal threshold to 3 min for all cell lines, which yielded proximal state fractions below 5% in auxin-treated cells (Fig. 2b), where closed states are not expected. We also examined variations of this temporal threshold to estimate the range of possible estimated proximal state fractions, frequencies and lifetimes (Extended Data Fig. 4f-h). To segment proximal states, we identified all time intervals with 3D anchor-anchor distances below the spatial threshold for durations exceeding the temporal threshold. For each distance time series, this yielded a binary time series, where 1 indicates the presence and 0 the absence of a proximal state. We then applied a rolling average over sliding time windows of duration equal to the temporal threshold to filter out the effect of brief distance fluctuations and to avoid fragmenting proximal states, as previously validated on polymer simulations[12]. Finally, we labelled as proximal states all timepoints with a resulting value above 0.5 (and non-proximal otherwise; Extended Data Fig. 4a). Obviously, this method cannot detect proximal states shorter than the temporal threshold (Extended Data Fig. 4d).

To segment proximal states in the dataset acquired at a higher imaging frequency ($\delta t = 9$ s; Extended Data Fig. 6f,i), we adjusted the percentile of the theoretical closed state distribution to ensure that the probability of observing a closed state for a given temporal threshold remained the same as in the original dataset ($\delta t = 30$ s). Specifically, for the low-frequency dataset, we applied a temporal threshold of 3 min (6 frames) and used a spatial threshold corresponding to the 95$^{th}$ percentile of the theoretical closed state distribution. This resulted in a probability of observing a closed state for 6 consecutive frames of $0.95^6 = 0.74$. To maintain the same probability in the high-frequency dataset with the same temporal threshold of 3 min (20 frames), we increased the spatial percentile to 98.5. This corresponds to the same probability of observing a closed state for 20

consecutive frames ($0.985^{20} = 0.74$). This adjustment led to higher spatial thresholds of 0.312, 0.292, and 0.343 $\mu$m for L2, Half TAD, and the Adjacent locus, respectively (Supplementary Table 2).

### Computing fraction, frequency and lifetime of proximal states

The fraction of proximal states reported in Fig. 2b was computed as: $\frac{N_{\text{proximal}}}{N_{\text{total}}}$, where $N_{\text{proximal}}$ denotes the number of timepoints in the proximal state and $N_{\text{total}}$ denotes the total number of timepoints.

The frequency of proximal states reported in Fig. 2c was computed as: $\frac{N_{\text{occ}}}{N_{\text{total}} * \delta t}$, where $N_{\text{occ}}$ is the number of proximal state intervals and $\delta t$ is the time interval between two acquired 3D image stacks.

The mean lifetime of proximal states and its confidence interval reported in Fig. 2d and Extended Data Fig. 4e were computed by fitting an exponential function to the histogram of proximal state durations (Extended Data Fig. 4d), taking into account censoring as in Gabriele *et al*[34].

### Estimation of loop state fractions

To estimate loop state fractions, we used a method previously described and validated on polymer simulations in which an analytical 3-state model is fitted to the distribution of anchor coordinate differences[12] (Extended Data Fig. 5a). Briefly, we assumed that the coordinate differences between TAD anchors $\delta v$ ($v \in \{x, y, z\}$) follow a normal distribution of mean 0 and variance $\sigma^2 + 2\sigma_v^2$, given by:

$$\mathrm{P}_v(\delta v; \sigma) = \frac{1}{(2\pi)^{\frac{1}{2}}(\sigma^2 + 2\sigma_v^2)^{\frac{1}{2}}} exp\left(-\frac{1}{2}\left(\frac{\delta v^2}{\sigma^2 + 2\sigma_v^2}\right)\right), \text{with } v \in \{x, y, z\}$$

where $\sigma_v$ is the localization precision for dimension $v$ and $\sigma$ depends on the loop state as described in the following.

In the proximal and open states, $\sigma$ is assumed to be constant and is called $\sigma_{\mathrm{proximal}}$ and $\sigma_{\mathrm{open}}$, respectively. For each cell line, we obtained the variances of coordinate differences $\sigma^2_{\mathrm{proximal}}$ and $\sigma^2_{\mathrm{open}}$ by fitting this Gaussian model to the distribution of anchor-anchor coordinate differences in the segmented proximal states and auxin-treated cells, respectively. For each distribution, the localization precision for dimension $v$ was set to the average localization precision:

$$\sigma_v = \frac{1}{N}\sum_{i=1}^{N}\sqrt{e^2_{i,r,v} + e^2_{i,g,v}}$$

where $N$ is the number of coordinate differences in the distribution, $e_{i,c,v}$ is the estimated localization precision for spot $i$ (see section 'Refining localizations and measuring localization precision'), and $c \in \{r, g\}$ indicates the color channel. In the extruding state, the distribution of coordinate differences is modelled as an integral over $\sigma^2$ varying from $\sigma^2_{\mathrm{proximal}}$ to $\sigma^2_{\mathrm{open}}$. This model assumes that the polymer is at equilibrium at each step of the extrusion process and that the anchors in the extruding state behave as if part of a shorter polymer in which the loop is absent[12]. The full analytical model reads:

$$\mathcal{P}_v(\delta v; \sigma) = A_{\mathrm{proximal}}\mathrm{P}_v\big(\delta v; \sigma_{\mathrm{proximal}}\big) + A_{\mathrm{open}}\mathrm{P}_v\big(\delta v; \sigma_{\mathrm{open}}\big) + A_{\mathrm{extrusion}}\int_{\sigma^2_{\mathrm{proximal}}}^{\sigma^2_{\mathrm{open}}}\mathrm{P}_v(\delta v; s)\,ds^2$$

where $A_{\mathrm{proximal}}, A_{\mathrm{extruding}}, A_{\mathrm{open}}$ are the three loop state fractions to be estimated. This model was fitted to the three distributions of coordinate differences ($\delta x, \delta y$ and $\delta z$) simultaneously.

We used polymer simulations to verify that the three loop states described above (open, extruding and proximal), were sufficient to accurately describe loop conformations. Specifically, our analytical model does not account for polymer relaxation following cohesin dissociation, which could potentially affect our quantifications of loop states. However, we found that polymer relaxation is rare (it occurred for 8% of closed states, which are only observed 0-1% of the time) and too transient relative to our imaging frequency (~1 min, corresponding to 1-2 imaging frames) to affect our conclusions (Extended Data Fig. 5d-f). Then, we evaluated our analytical model using polymer simulations as ground truth. We found that our method accurately estimated open states

(absolute errors of 0%) in most conditions and especially those consistent with experiments (Fig. 5c and Extended Data Fig. 5g). Proximal states were slightly overestimated at the expense of extruding states (absolute error of ~18%; Extended Data Fig. 5g), reflecting the broader definition of proximal states compared to closed states (Extended Data Fig. 10).

For proximal states and auxin-treated cells, we defined two independent datasets. The first set was used to estimate $\sigma^2_{\mathrm{proximal}}$ and $\sigma^2_{\mathrm{open}}$, while the second set was used to estimate loop state fractions. For untreated cells, we used a single dataset to estimate loop state fractions. To estimate ranges of loop state fractions, we used bootstrapping whereby we randomly drew time series (and not single timepoints) with replacement from the original dataset, using 100% of the available time series.

**Model-free estimation of the maximal fraction of open states**

As additional evidence for our claim that TADs constantly undergo extrusion, we provide a model-free analysis showing that open states are rare or nearly absent. Unlike the 3-state model described above, this analysis does not use any model but relies only on the experimentally measured 3D anchor-anchor distance distributions in presence or absence of auxin. Specifically, we considered the distance distribution measured in auxin-treated cells -where cohesin is depleted and the anchors are expected to be mostly in the open state- and asked what proportion of distances measured in the untreated cells could possibly arise from the auxin-treated distance distribution (Extended Data Fig. 5c). To do this, we weighted the “+ Auxin” distribution by a multiplicative coefficient between 0 and 1 that was determined to be the largest possible such that 95% of the distance bins from the weighted distribution fit under the “untreated” distribution. We assumed that the untreated distance histogram is a sum of two distributions: the distribution of distances in open states and the distribution in non-open states. This assumption leads to the estimation of the multiplicative coefficient as the upper limit to the fraction of open states in untreated cells, since extruding states can exhibit distances similar to the open state. This analysis led to an upper bound for the multiplicative coefficient and hence to maximal

open state fractions in untreated cells of 18%, 4% and 5% for L1, L2 and T1, respectively (Extended Data Fig. 5c). This analysis confirms without any model or assumption on loop states that open states are rare or nearly absent.

## Estimation of closing rate

To estimate the closing rate, we adapted a method previously validated on polymer simulations[12]. We aligned time series such that the segmented proximal states coincide at $t = 0$ and computed the ensemble mean squared anchor-anchor distance (EMSAAD) $\langle\langle R^2 \rangle\rangle(t)$ from these aligned time series:

$$\langle\langle R^2 \rangle\rangle(t) = \left( \sum_{j=1}^{n} w_j(t) \right)^{-1} \sum_{j=1}^{n} w_j(t) \left( d_j^2(t) - e_{d,j}^2(t) \right)$$

where $d_j^2(t), e_{d,j}^2(t)$ and $w_j(t)$ are the squared anchor-anchor distance, the squared localization precision and the weight for the track $j \in \{1, \dots, n\}$ at time $t$, respectively (see above 'Refining localizations and measuring localization precision' and 'Scoring of localization precision'). This weighting allows us to reduce the influence of distances associated with poor precisions $e_d$ on the closing rate estimation. We also computed the weighted standard error of the EMSAAD:

$$\sigma_{\text{EMSAAD}}(t) = \sqrt{\frac{\sum_{j=1}^{n} w_j(t) \left( d_j^2(t) - e_{d,j}^2(t) - \langle\langle R^2 \rangle\rangle(t) \right)^2}{n \sum_{j=1}^{n} w_j(t)}}$$

We fitted the EMSAAD to the following model $f$, a piecewise linear curve defined by three parameters $\{t_{\text{extr}}, R_{\text{plateau}}^2, R_{\text{int}}^2\}$:

$$f(t) = \begin{cases} R_{\text{plateau}}^2 & if\ t \le t_{\text{extr}} \\ \dfrac{R_{\text{plateau}}^2 - R_{\text{int}}^2}{t_{\text{extr}}} t + R_{\text{int}}^2 & if\ t > t_{\text{extr}} \end{cases}$$

where $R_{\text{plateau}}^2$ is the averaged mean squared distance before we can detect the influence of incoming cohesin complex(es) on anchor-anchor distances, $t_{\text{extr}}$ is the time at which distances start to decrease due to the action of cohesin complexes that determine the shortest 1D path between anchors, and $R_{\text{int}}^2$ is set to the squared distance at time $t = 0$

(Fig. 3c). We fitted the model $f$ to either experimental or simulated EMSAAD by minimizing the mean squared error $L = \sum_{t=-t_w}^{0} \left( \langle\langle R^2 \rangle\rangle(t) - f(t) \right)^2$ over the time interval $[-t_w, 0]$ preceding the proximal states (Fig. 3d and 5d,e), where $t_w$ is the total time window and was set to $t_w = 25$ min. Because $f(t)$ is not differentiable at $t = t_{\mathrm{extr}}$, $R^2_{\mathrm{plateau}}$ was estimated by least-squares regression for all discrete values of $t_{\mathrm{extr}}$ in $]-t_w, 0[$ (with $R^2_{\mathrm{plateau}}$ initialized at the mean value of EMSAAD from $t_w$ to $t_{\mathrm{extr}}$) and we finally chose the pair of parameters $(t_{\mathrm{extr}}, R^2_{\mathrm{plateau}})$ providing the smallest $L$.

Since our closing rate estimation focuses only on proximal states, we applied a more stringent spatial threshold to ensure a more conservative segmentation of proximal states and minimize inaccuracies from ill-segmented time series[12]. Specifically, we defined the spatial thresholds based on the 75$^{\mathrm{th}}$ percentile of the theoretical closed state distribution, instead of the 95$^{\mathrm{th}}$ percentile (see section 'Proximal state segmentation'). This resulted in smaller distance thresholds of 0.136, 0.146, 0.159 and 0.143 $\mu$m for L1, L2, T1 and Half TAD, respectively. We used a temporal window of $t_w = 25$ min. We ignored proximal states if an earlier proximal state occurred within the time window $t_w$. This filtering and the size of the fitting window ($t_w$) did not affect the estimation of closing rates, as long as $t_w$ was sufficiently long to observe a plateau in squared distances (Extended Data Fig. 7c). The fitting of the EMSAAD to the model $f$ was performed only when at least 15 time series could be aligned, and the closing rate was computed only if the linear decrease was supported by at least three timepoints. Otherwise, we considered the averaged time series to be consistent with a constant model of distances and did not estimate the closing rate (Fig. 5e and Extended Data Figs. 7a,b and 9e-g).

As negative controls for the closing rate estimation, we randomly shuffled time points within individual time series. We then segmented proximal states and estimated the closing rate using the exact same procedure as for the original (unshuffled) data. Time series were randomized for each bootstrap sample.

Using polymer simulations, we found that motor speeds of 0.5 kb/s or more were consistent with constant distances. These high speeds did not allow us to estimate closing rates at our imaging frequency of $\delta t = 30\,s$ (Fig. 5e and Extended Data Fig. 9e). By

contrast, increasing the sampling frequency by 3.3-fold (*i.e.* $\delta t = 9\ s$) in simulations allowed to detect a linear decrease in nearly 100% of bootstrap samples at 0.5 kb/s *vs* only 0-10% for L2, T1 and Half TAD, and for 86% in L1, at the original frequency (Extended Data Fig. 9e-g), confirming that the original sampling rate was not sufficient to estimate closing rates at high motor speeds. However, since we were in fact able to detect the expected linear decrease in experiments, we can rule out speeds of 0.5 kb/s or more (Fig. 5d and Extended Data Fig. 7b).

## 3D polymer simulations of loop extrusion

### Molecular dynamics simulations

Polymer motion was simulated with Langevin dynamics in LAMMPS (vNov16)[35]. The polymer was modelled as a freely jointed chain, where consecutive monomers (beads) were connected by a harmonic bond with a potential $E_{\text{bond}} = 30(r-1)^2$, where $r$ is the distance between bead centers. Additional harmonic bonds were created transiently between non-consecutive beads to simulate loop extrusion (see subsection 'Loop extrusion modeling' below). We simulated chromatin segments of 2.6 Mb centered on each of the domains studied experimentally, using 1,300 beads, each representing 2 kb of DNA. We used fixed boundary conditions and confined the polymer to a sphere of radius 24 bead diameters with a potential $E_{\text{wall}} = 4\left[\left(\frac{\sigma}{R}\right)^{12} - \left(\frac{\sigma}{R}\right)^{6}\right]$ for $R < R_c$, where $R$ is the distance between the confining sphere and the center of a bead, $\sigma$ is a size factor set to 0.5 bead diameter and $R_c$ is the cutoff distance, which was also set to 0.5 bead diameter. This led to a volume occupancy ratio of 10% in agreement with the experimentally measured chromatin density of 10-15% in the nucleus[36–38].

We converted simulation units into physical units by comparing the 2-point MSD curves of simulations without loop extrusion to experimental data from auxin-treated cells for each genomic locus, as follows. First, we determined the physical bead size for which the 2-point MSD plateaued at the same level in simulations and experiments, using a bead size of 2 kb. This yielded a bead diameter of 45 nm and thus a DNA compaction of

44 bp/nm consistent with previous estimates of 18-66 bp/nm[29,31,32]. Second, we converted simulation time steps into time units such that the simulated and experimental 2-point MSD reached half of the plateau for the same time lag. This led to a 3 s interval for 2,000 simulation timesteps.

The polymer was first equilibrated for $10^6$ simulations steps, after which its radius of gyration and end-to-end distances were stabilized. Then, we ran 400 extrusion steps at 27 kb/s and 400 extrusion steps at 5 kb/s to renew the pool of already loaded cohesin complexes. Finally, 1,300 extrusion steps were performed at the defined motor speed before recording conformation snapshots during $4.8 \times 10^6$ simulation steps, corresponding to 2 hours of physical time, as in the experimental live-cell imaging data. Simulations were run for a total of $15 \times 10^6$ simulation timesteps representing 11 hours to 5 days of computation time per simulation. For each combination of cohesin parameters, we generated 50 independent simulations. For the analysis of closing rates in Fig. 5e and Extended Data Fig. 9e-g, we generated 600 independent simulations for each parameter combination.

## Loop extrusion modeling

Cohesin-mediated loop extrusion was simulated by the creation and destruction of harmonic bonds between non-consecutive polymer beads. In absence of obstacles, loop extrusion was bi-directional, but extrusion switched to uni-directional when the cohesin was blocked by a CTCF site on one side[39]. Cohesin complexes were loaded at random positions along the polymer and at a rate defined by the cohesin density divided by its residence time, therefore ensuring constant cohesin density. Cohesin complexes detached at a rate defined by the cohesin residence time, which was assumed to follow an exponential distribution. In light of recent experimental evidence, we allowed cohesin complexes to traverse each other[40–42].

Cohesin motor speed was specified by the number of simulation time steps separating the creation of a new harmonic bond between non-consecutive beads (and the destruction of an existing bond). Upon encountering a convergently oriented CTCF site,

extruding complexes were stopped with a probability of 50% and else proceeded unimpeded, based on the estimated 50% occupancy of CTCF sites by the CTCF protein[43]. Cohesin complexes were allowed to stall at CTCF sites for a duration drawn from an exponential probability distribution with a mean time (corresponding to the CTCF residence time) that was defined specifically for each CTCF site based on CTCF ChIP-Seq data (see section 'Modeling CTCF residence on chromatin'). At the extremities of the polymer, we assumed an infinite CTCF residence time and a 50% probability of stalling cohesin, in order to compensate for the fact that our simulation did not account for cohesin complexes moving into the chromatin domain from the other parts of the chromosome.

**Modeling CTCF residence on chromatin**

We assigned a specific residence time to each CTCF site based on the corresponding ChIP-Seq peak. We defined a 2.6 Mb region (corresponding to the length of the simulated polymer) centered on the TAD of each genomic region and retrieved CTCF sites mapped on CTCF ChIP-Seq peaks. For each CTCF site, we used the CTCF peak fold enrichment computed by MACS2, which takes into account the local background, as a measure of CTCF residence time. We computed the median fold enrichment for all peaks identified across the entire genome and used it to normalize the CTCF fold enrichment within the studied genomic region. We thus assumed that the affinity of the binding site linearly scales with ChIP-Seq fold enrichment, as predicted by sequence to affinity models[44]. We then used previous estimations of CTCF residence time of 1-4 min in mESCs[45,46] to convert the median genome-wide ChIP-Seq fold enrichment to a CTCF residence time of 2.5 min. Therefore, CTCF sites with fold enrichments above the genome-wide median had residence times longer than 2.5 min (Fig. 4b). Because CTCF sites are defined as 20 bp-motifs, multiple binding sites and ChIP-Seq peaks can be present within a single 2 kb polymer bead. If these CTCF sites shared the same orientation, we summed the peak fold enrichments. In the few cases where one CTCF ChIP-Seq peak overlapped two binding sites of opposite orientations, the CTCF residence time associated with each orientation was adjusted based on the relative p-value of the two CTCF sites.

Note that we chose to attribute differences between CTCF ChIP-Seq peaks to differences in CTCF residence time (*i.e.* dissociation rate), rather than to CTCF association rate. While bulk ChIP-Seq cannot differentiate between the contributions of the association rate $k_{on}$ and the dissociation rate $k_{off}$, it was found that changes in the binding site sequence primarily affect $k_{off}$ rather than $k_{on}$[47]. Therefore, differences between ChIP-Seq peaks are more likely to reflect variations in CTCF residence time, rather than variations in CTCF association rate.

**Comparison of simulations to experiments**

For each of the experimental genomic regions, we explored a simulation parameter space wherein the cohesin density ranged from 1 to 40 $Mb^{-1}$, the cohesin residence time from 2 to 33 min, and considered cohesin motor speeds of 0.25 and 1 kb/s (Fig. 5a). This parameter space encompassed current experimentally or computationally estimated ranges of cohesin densities (4-32 cohesin per Mb[34,43,46,48,49]), cohesin residence times (3-25 min[45,49–54]), and the 0.5-1 kb/s cohesin motor speeds estimated *in vitro*[55,56], in addition to the rough estimates of 0.2-1.3 kb/s obtained *in vivo* when dividing the cohesin residence time by the median loop length of 230 kb (Extended Data Fig. 1d). We did not explore cohesin residence times longer than 33 min because the corresponding processivities (~1,000 kb at 1 kb/s, Extended Data Fig. 8g) had the same order of magnitude as the length of the simulated polymer (2,600 kb) and simulations could be affected by edge effects.

One-dimensional (1D) simulations of cohesin-mediated loop extrusion (see section 'Loop extrusion modeling' above) were used to define temporary bonds between polymer beads, which were used to simulate 3D polymer motions (section 'Molecular dynamics simulations' above). From the 3D simulations, we computed the 3D distance between beads located near, but not at, TAD anchors, consistent with the genomic distance separating the center of repeat arrays and the CTCF site defining each anchor (3.9 to 8 kb depending on the genomic region, Supplementary Table 2). To account for finite localization precision of fluorescent spots in experimental time series, we added random

Gaussian localization errors to the exact coordinates from polymer simulations, using standard deviation given by the Cramér-Rao lower bound estimated from the experiments[24] (see subsection 'Refining localizations and measuring localization precision' above). To model the progressive decrease in localization precision over time due to fluorophore photobleaching, we fitted a linear function to the mean experimental localization precisions $\langle e_{c,v}(t) \rangle$ in $x$, $y$ and $z$ as function of time, for each color channel, and for each cell line. We then used this linear function to add time-dependent localization errors to the simulated exact coordinates. Finally, we sampled simulations to 1 frame per 30 seconds and 241 frames in total, as in live-cell imaging experiments. The same spatial and temporal thresholds used to segment proximal states in experimental tracking data were used for simulated time series. Unless otherwise stated, we used simulations including noise from localization errors, finite genomic length between CTCF sites and fluorescent reporters and at a temporal resolution of 30 s per frame.

To build contact maps from polymer simulations, we sampled 100 conformations from each of the 50 independent simulations generated for each set of parameters. We used a capture radius of 1 bead (*i.e.* 45 nm) to model Micro-C maps and define contacts. This threshold is below the 100-150 nm capture radius estimated from comparison of spatial distance maps to Hi-C[57,58]. Indeed, Micro-C is expected to have a shorter capture radius than Hi-C[59,60]. We restricted our analysis to contact maps spanning 1.1 times the length of the studied TAD.

To compare polymer simulations with experiments, we considered the following six metrics: anchor-anchor 3D distance distributions, the p(s) curve from contact maps, 2-point MSD curves, proximal state fraction and frequency distributions obtained from bootstrapping, and proximal state lifetime. Note that simulated and experimental p(s) were normalized to a value of 1 at 20 kb (Extended Data Fig. 9h). Likewise, for the 2-point MSD curves we normalized the first time lag value to 1 and considered values for a maximum time lag of 300 seconds (Extended Data Fig. 9i).

For each quantity and set of simulated parameters, the deviation of polymer simulations from the experimental data was computed as:

$$\text{Deviation} = \frac{\sum_i (y_{i,s} - y_{i,e})^2}{\sum_i (y_{i,s}^2 + y_{i,e}^2)}$$

where $i$ represents each sample, and $y_s$ and $y_e$ are the simulated and experimental values, respectively. The computed deviation values are dimensionless and lie between 0 and 1. To compute the overall deviation of simulations from experiments, we summed the deviations from all metrics (Extended Data Fig. 9a). Simulations with the smallest deviations were considered the best matches.

Genomic processivity was computed as the average genomic distance that cohesin complexes reached on the simulated polymer, taking into account stalling at CTCF sites (Extended Data Fig. 8g). The cohesin loading rate was computed as: $\text{loading rate} = \frac{\text{density}}{\text{residence time}}$ (Extended Data Fig. 8i).

## Characterization of proximal and closed states

We assessed how segmented proximal states relate to closed states using polymer simulations with perfectly known ground truth. We recall that closed states are strictly defined as direct interactions between the two TAD anchors, excluding cases where the anchors are indirectly connected *via* multiple loops. Proximal states, however, are defined as time intervals longer than the temporal threshold, during which all distances fall below the spatial threshold.

Based on simulations, we found that closed state timepoints were generally identified as proximal states, since 79%, 91%, and 91% of single timepoints in the closed state were labelled as proximal for L1, L2 and T1, respectively (Extended Data Fig. 10a). Comparing temporal intervals instead of single timepoints led to a similar conclusion, since 78%, 95%, and 100% of closed state intervals intersected a single proximal state interval for L1, L2 and T1, respectively (Extended Data Fig. 10b). Proximal state intervals intersecting closed state intervals generally included the closed state interval entirely and extended before and after its starting and ending timepoints, respectively (Fig. 6c), thus proximal states had longer durations than closed states (Extended Data Fig. 10c). The closed state intervals that did not intersect a proximal state interval were generally shorter than the

3 min temporal threshold used for segmentation (Extended Data Fig. 10d). By contrast, 94%, 98% and 99% of proximal states did not intersect a closed state in L1, L2 and T1, respectively (Extended Data Fig. 10e). However, these proximal state intervals occurred toward the end of the extrusion phase, when the unextruded genomic distance between anchors was already small (Fig. 6c and Extended Data Fig. 10f). Because multiple cohesin complexes can simultaneously extrude TADs, anchors can be in spatial proximity even without being connected by a unique loop in a closed state (Fig. 6c,d). For this reason, we found that proximal states involved on average 1.9-3.2 loops, which concomitantly connected TAD anchors (Extended Data Fig. 10g).

## Supplementary Tables

**Supplementary Table 1: Overview of imaging experiments.**

All statistics are from quality-filtered datasets.

**Supplementary Table 2: Parameters used for proximal state segmentation.**

NA: Not analyzed.

**Supplementary Table 3: List of primers used in this study**

Abbreviations: Neo: Neomycin resistance gene, Bsd: Blasticidin, TK: thymidine kinase.

**Supplementary Table 4: List of plasmids used in this study.**

Abbreviations: Puro: Puromycin, GB1: B1 domain of Streptococcal protein G, NLS: Nuclear localization signal, HA5/3: homology arms 5'/3', Neo: Neomycin resistance gene, Bsd: Blasticidin, TK: thymidine kinase.

## Supplementary Videos

**Supplementary Video 1: Live-cell imaging of L1 TAD anchors.**

Live-cell imaging of L1 TAD anchors from cells left untreated (top) or after a 2-hour auxin-treatment (bottom). Timestamp indicates time as minutes:seconds.

**Supplementary Video 2: 1D simulations of each genomic region.**

A cohesin density of 12 $Mb^{-1}$, residence time of 22 min and motor speed of 0.25 kb/s were assumed. Black dotted lines indicate the anchors of each TAD (or fluorescent reporter for the half TAD control) and red lines indicate loops involved in the shortest 1D path between anchors. Green and blue arrows indicate the orientation of CTCF sites, while their height indicates the associated CTCF residence time. Timestamp indicates time as hours:minutes.

# Supplementary Figure 1

**a Fraction of cells in G1 with or without a 3-hour auxin treatment**

1) Remove large cells with high granularity

2) Discard doublets and cells in M phase

3) Retrieve distribution of propidium iodide area from >12,000 cells

4) Fit the Dean Jett Fox model without constraint to obtain fraction of cells in G1

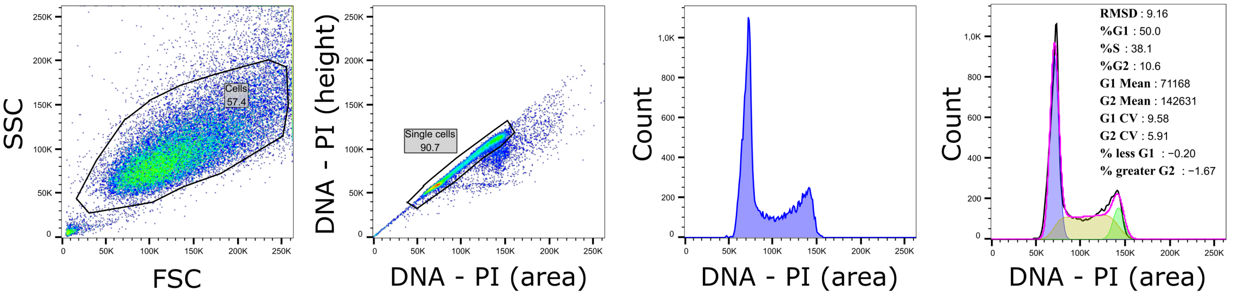


Gates were the same for each biological replicate.

**b Fraction of polyploid cells (at least triploid)**

1) Remove large cells with high granularity

2) Quantify single polyploid cells

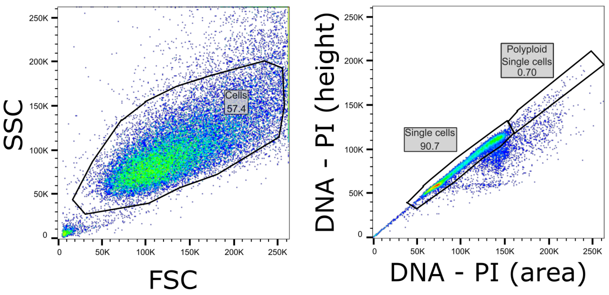


Gates were the same for each biological replicate.

## Supplementary Figure 1: Flow cytometry gating strategy

**a,** Gating and fitting strategy to define the fraction of cells in G1. **b,** Gating strategy to define the fraction of polyploid cells.

## References


1. Rao, S. S. P. *et al.* Cohesin Loss Eliminates All Loop Domains. *Cell* **171**, 305-320.e24 (2017).
2. Durand, N. C. *et al.* Juicer provides a one-click system for analyzing loop-resolution Hi-C experiments. *Cell Syst* **3**, 95–98 (2016).
3. Babraham Bioinformatics - FastQC A Quality Control tool for High Throughput Sequence Data. https://www.bioinformatics.babraham.ac.uk/projects/fastqc/.
4. Langmead, B. & Salzberg, S. L. Fast gapped-read alignment with Bowtie 2. *Nat Methods* **9**, 357–359 (2012).
5. Kent, W. J., Zweig, A. S., Barber, G., Hinrichs, A. S. & Karolchik, D. BigWig and BigBed: enabling browsing of large distributed datasets. *Bioinformatics* **26**, 2204–2207 (2010).
6. Amemiya, H. M., Kundaje, A. & Boyle, A. P. The ENCODE Blacklist: Identification of Problematic Regions of the Genome. *Sci Rep* **9**, 9354 (2019).
7. Zhang, Y. *et al.* Model-based Analysis of ChIP-Seq (MACS). *Genome Biology* **9**, R137 (2008).
8. Grant, C. E., Bailey, T. L. & Noble, W. S. FIMO: scanning for occurrences of a given motif. *Bioinformatics* **27**, 1017–1018 (2011).
9. Greenwald, W. W. *et al.* Pgltools: a genomic arithmetic tool suite for manipulation of Hi-C peak and other chromatin interaction data. *BMC Bioinformatics* **18**, 207 (2017).
10. Fishilevich, S. *et al.* GeneHancer: genome-wide integration of enhancers and target genes in GeneCards. *Database* **2017**, bax028 (2017).
11. Labun, K. *et al.* CHOPCHOP v3: expanding the CRISPR web toolbox beyond genome editing. *Nucleic Acids Research* **47**, W171–W174 (2019).
12. Sabaté, T., Lelandais, B., Bertrand, E. & Zimmer, C. Polymer simulations guide the detection and quantification of chromatin loop extrusion by imaging. *Nucleic Acids Research* **51**, 2614–2632 (2023).

13. Brandão, H. B., Gabriele, M. & Hansen, A. S. Tracking and interpreting long-range chromatin interactions with super-resolution live-cell imaging. *Current Opinion in Cell Biology* **70**, 18–26 (2021).
14. Gupta, P., Lavagnolli, T., Mira-Bontenbal, H., Fisher, A. G. & Merkenschlager, M. Cohesin's role in pluripotency and reprogramming. *Cell Cycle* **15**, 324–330 (2016).
15. Tinevez, J.-Y. *et al.* TrackMate: An open and extensible platform for single-particle tracking. *Methods* **115**, 80–90 (2017).
16. Mikolajczyk, K. & Schmid, C. A performance evaluation of local descriptors. *IEEE Transactions on Pattern Analysis and Machine Intelligence* **27**, 1615–1630 (2005).
17. Zhang, B., Zerubia, J. & Olivo-Marin, J.-C. Gaussian approximations of fluorescence microscope point-spread function models. *Appl. Opt., AO* **46**, 1819–1829 (2007).
18. Lowe, D. G. Distinctive Image Features from Scale-Invariant Keypoints. *International Journal of Computer Vision* **60**, 91–110 (2004).
19. Jaqaman, K. *et al.* Robust single-particle tracking in live-cell time-lapse sequences. *Nat Methods* **5**, 695–702 (2008).
20. Ershov, D. *et al.* TrackMate 7: integrating state-of-the-art segmentation algorithms into tracking pipelines. *Nat Methods* **19**, 829–832 (2022).
21. Chenouard, N. *et al.* Objective comparison of particle tracking methods. *Nature Methods* **11**, 281–289 (2014).
22. Schindelin, J. *et al.* Fiji: an open-source platform for biological-image analysis. *Nat Methods* **9**, 676–682 (2012).
23. Matsuda, A., Schermelleh, L., Hirano, Y., Haraguchi, T. & Hiraoka, Y. Accurate and fiducial-marker-free correction for three-dimensional chromatic shift in biological fluorescence microscopy. *Sci Rep* **8**, 7583 (2018).
24. Ober, R. J., Ram, S. & Ward, E. S. Localization Accuracy in Single-Molecule Microscopy. *Biophysical Journal* **86**, 1185–1200 (2004).
25. Haering, C. H., Löwe, J., Hochwagen, A. & Nasmyth, K. Molecular Architecture of SMC Proteins and the Yeast Cohesin Complex. *Molecular Cell* **9**, 773–788 (2002).
26. Kaur, P. *et al.* High-speed AFM imaging reveals DNA capture and loop extrusion dynamics by cohesin-NIPBL. *Journal of Biological Chemistry* **299**, 105296 (2023).

27. Dekker, C., Haering, C. H., Peters, J.-M. & Rowland, B. D. How do molecular motors fold the genome? *Science* **382**, 646–648 (2023).
28. Barth, R. *et al.* Testing pseudotopological and nontopological models for SMC-driven DNA loop extrusion against roadblock-traversal experiments. *Sci Rep* **13**, 8100 (2023).
29. Arbona, J.-M., Herbert, S., Fabre, E. & Zimmer, C. Inferring the physical properties of yeast chromatin through Bayesian analysis of whole nucleus simulations. *Genome Biology* **18**, 81 (2017).
30. Dekker, J., Rippe, K., Dekker, M. & Kleckner, N. Capturing Chromosome Conformation. *Science* **295**, 1306–1311 (2002).
31. Lesage, A., Dahirel, V., Victor, J.-M. & Barbi, M. Polymer coil–globule phase transition is a universal folding principle of Drosophila epigenetic domains. *Epigenetics & Chromatin* **12**, 28 (2019).
32. Dekker, J. Mapping in Vivo Chromatin Interactions in Yeast Suggests an Extended Chromatin Fiber with Regional Variation in Compaction. *Journal of Biological Chemistry* **283**, 34532–34540 (2008).
33. Ringrose, L., Chabanis, S., Angrand, P., Woodroofe, C. & Stewart, A. F. Quantitative comparison of DNA looping in vitro and in vivo: chromatin increases effective DNA flexibility at short distances. *The EMBO Journal* **18**, 6630–6641 (1999).
34. Gabriele, M. *et al.* Dynamics of CTCF- and cohesin-mediated chromatin looping revealed by live-cell imaging. *Science* **376**, 496–501 (2022).
35. Thompson, A. P. *et al.* LAMMPS - a flexible simulation tool for particle-based materials modeling at the atomic, meso, and continuum scales. *Computer Physics Communications* **271**, 108171 (2022).
36. Woringer, M., Darzacq, X. & Izeddin, I. Geometry of the nucleus: a perspective on gene expression regulation. *Current Opinion in Chemical Biology* **20**, 112–119 (2014).
37. Rippe, K. Dynamic organization of the cell nucleus. *Current Opinion in Genetics & Development* **17**, 373–380 (2007).
38. Dekker, J. & Misteli, T. Long-Range Chromatin Interactions. *Cold Spring Harb Perspect Biol* **7**, a019356 (2015).

39. Davidson, I. F. *et al.* CTCF is a DNA-tension-dependent barrier to cohesin-mediated loop extrusion. *Nature* **616**, 822–827 (2023).
40. Kim, E., Kerssemakers, J., Shaltiel, I. A., Haering, C. H. & Dekker, C. DNA-loop extruding condensin complexes can traverse one another. *Nature* **579**, 438–442 (2020).
41. Brandão, H. B., Ren, Z., Karaboja, X., Mirny, L. A. & Wang, X. DNA-loop-extruding SMC complexes can traverse one another in vivo. *Nat Struct Mol Biol* **28**, 642–651 (2021).
42. Banigan, E. J., van den Berg, A. A., Brandão, H. B., Marko, J. F. & Mirny, L. A. Chromosome organization by one-sided and two-sided loop extrusion. *eLife* **9**, e53558 (2020).
43. Cattoglio, C. *et al.* Determining cellular CTCF and cohesin abundances to constrain 3D genome models. *eLife* **8**, e40164 (2019).
44. Riley, T. R., Lazarovici, A., Mann, R. S. & Bussemaker, H. J. Building accurate sequence-to-affinity models from high-throughput in vitro protein-DNA binding data using FeatureREDUCE. *eLife* **4**, e06397 (2015).
45. Hansen, A. S., Pustova, I., Cattoglio, C., Tjian, R. & Darzacq, X. CTCF and cohesin regulate chromatin loop stability with distinct dynamics. *Elife* **6**, e25776 (2017).
46. Brunner, A. *et al.* Quantitative imaging of loop extruders rebuilding interphase genome architecture after mitosis. *Journal of Cell Biology* **224**, e202405169 (2025).
47. Khamis, H., Rudnizky, S., Melamed, P. & Kaplan, A. Single molecule characterization of the binding kinetics of a transcription factor and its modulation by DNA sequence and methylation. *Nucleic Acids Research* **49**, 10975–10987 (2021).
48. Fudenberg, G. *et al.* Formation of Chromosomal Domains by Loop Extrusion. *Cell Reports* **15**, 2038–2049 (2016).
49. Mach, P. *et al.* Cohesin and CTCF control the dynamics of chromosome folding. *Nat Genet* **54**, 1907–1918 (2022).
50. Wutz, G. *et al.* Topologically associating domains and chromatin loops depend on cohesin and are regulated by CTCF, WAPL, and PDS5 proteins. *The EMBO Journal* **36**, 3573–3599 (2017).

51. Holzmann, J. *et al.* Absolute quantification of cohesin, CTCF and their regulators in human cells. *eLife* **8**, e46269 (2019).
52. Wutz, G. *et al.* ESCO1 and CTCF enable formation of long chromatin loops by protecting cohesinSTAG1 from WAPL. *Elife* **9**, e52091 (2020).
53. Gerlich, D., Koch, B., Dupeux, F., Peters, J.-M. & Ellenberg, J. Live-Cell Imaging Reveals a Stable Cohesin-Chromatin Interaction after but Not before DNA Replication. *Current Biology* **16**, 1571–1578 (2006).
54. Kueng, S. *et al.* Wapl Controls the Dynamic Association of Cohesin with Chromatin. *Cell* **127**, 955–967 (2006).
55. Davidson, I. F. *et al.* DNA loop extrusion by human cohesin. *Science* **366**, 1338–1345 (2019).
56. Kim, Y., Shi, Z., Zhang, H., Finkelstein, I. J. & Yu, H. Human cohesin compacts DNA by loop extrusion. *Science* **366**, 1345–1349 (2020).
57. Takei, Y. *et al.* Integrated spatial genomics reveals global architecture of single nuclei. *Nature* **590**, 344–350 (2021).
58. Su, J.-H., Zheng, P., Kinrot, S. S., Bintu, B. & Zhuang, X. Genome-Scale Imaging of the 3D Organization and Transcriptional Activity of Chromatin. *Cell* **182**, 1641-1659.e26 (2020).
59. Krietenstein, N. *et al.* Ultrastructural Details of Mammalian Chromosome Architecture. *Molecular Cell* **78**, 554-565.e7 (2020).
60. Hsieh, T.-H. S. *et al.* Resolving the 3D Landscape of Transcription-Linked Mammalian Chromatin Folding. *Molecular Cell* **78**, 539-553.e8 (2020).
