# Supplementary material for: Uniform dynamics of cohesin-mediated loop extrusion in living human cells
Source: Nat Genet. 2025 Nov 14;57(12):3152–64. doi: 10.1038/s41588-025-02406-9 (PMC12695666; doi:10.1038/s41588-025-02406-9)
Supplement: Supplementary file 2 — Reporting Summary [file 41588_2025_2406_MOESM2_ESM.pdf]

nature portfolio

Corresponding author(s): Thomas Sabaté
Edouard Bertrand
Christophe Zimmer

Last updated by author(s): Sep 30, 2025

Reporting Summary

Nature Portfolio wishes to improve the reproducibility of the work that we publish. This form provides structure for consistency and transparency in reporting. For further information on Nature Portfolio policies, see our Editorial Policies and the Editorial Policy Checklist.

Statistics

For all statistical analyses, confirm that the following items are present in the figure legend, table legend, main text, or Methods section.

n/a	Confirmed	
☐	☒	The exact sample size (*n*) for each experimental group/condition, given as a discrete number and unit of measurement
☐	☒	A statement on whether measurements were taken from distinct samples or whether the same sample was measured repeatedly
☐	☒	The statistical test(s) used AND whether they are one- or two-sided<br>*Only common tests should be described solely by name; describe more complex techniques in the Methods section.*
☐	☒	A description of all covariates tested
☐	☒	A description of any assumptions or corrections, such as tests of normality and adjustment for multiple comparisons
☐	☒	A full description of the statistical parameters including central tendency (e.g. means) or other basic estimates (e.g. regression coefficient) AND variation (e.g. standard deviation) or associated estimates of uncertainty (e.g. confidence intervals)
☐	☒	For null hypothesis testing, the test statistic (e.g. *F*, *t*, *r*) with confidence intervals, effect sizes, degrees of freedom and *P* value noted<br>*Give P values as exact values whenever suitable.*
☒	☐	For Bayesian analysis, information on the choice of priors and Markov chain Monte Carlo settings
☒	☐	For hierarchical and complex designs, identification of the appropriate level for tests and full reporting of outcomes
☒	☐	Estimates of effect sizes (e.g. Cohen's *d*, Pearson's *r*), indicating how they were calculated

Our web collection on statistics for biologists contains articles on many of the points above.

Software and code

Policy information about availability of computer code

Data collection	For Western Blot imaging, we used the Chemidoc MP Imaging system (Bio-Rad).. For FACS, we used a Miltenyi MACSQuant Analyzer 10 Flow Cytometer with the 488 nm laser and a 692/75 nm band pass filter. Time lapse image acquisition was performed with an inverted microscope (Nikon) coupled to the Dragonfly spinning disk (Andor) using a 100X Plan Apo 1.45 NA oil immersion objective.
Data analysis	ChopChop v3, ImageJ v2.14, Labkit v0.4, TrackMate v7, FlowJo v10, FastQC v0.12.1, Cutadapt v0.6.10, HiC-Pro v3.1.0, Bowtie2 v2.2.6.2, Juicer 1.19.02, FIMO v5.3.0, pgltools v2.7.1, MACS v2.1, Chromagnon v0.94, LAMMPS Nov16, Python v3.8+.<br>The code (v095) used to generate polymer simulations, process live-cell microscopy images, quantify the distance between TAD anchors, and process the Micro-Capture C data is available at: https://github.com/imodpasteur/Sabate_et_al_TAD_Anchors and on Zenodo: 10.5281/zenodo.16949930.

For manuscripts utilizing custom algorithms or software that are central to the research but not yet described in published literature, software must be made available to editors and reviewers. We strongly encourage code deposition in a community repository (e.g. GitHub). See the Nature Portfolio guidelines for submitting code & software for further information.

Data

Policy information about availability of data

All manuscripts must include a data availability statement. This statement should provide the following information, where applicable:

- Accession codes, unique identifiers, or web links for publicly available datasets
- A description of any restrictions on data availability
- For clinical datasets or third party data, please ensure that the statement adheres to our policy

Capture Micro-C data have been uploaded to the Gene Expression Omnibus (GEO) under accession GSE273257. This paper analyzed existing, publicly available Hi-C, ChIP-Seq and PRO-Seq data from GEO under accession GSE104334. Raw and quality-filtered distance time series are available on Zenodo94 at: 10.5281/zenodo.16949930.

Research involving human participants, their data, or biological material

Policy information about studies with human participants or human data. See also policy information about sex, gender (identity/presentation), and sexual orientation and race, ethnicity and racism.

Reporting on sex and gender	*Use the terms sex (biological attribute) and gender (shaped by social and cultural circumstances) carefully in order to avoid confusing both terms. Indicate if findings apply to only one sex or gender; describe whether sex and gender were considered in study design; whether sex and/or gender was determined based on self-reporting or assigned and methods used. Provide in the source data disaggregated sex and gender data, where this information has been collected, and if consent has been obtained for sharing of individual-level data; provide overall numbers in this Reporting Summary. Please state if this information has not been collected. Report sex- and gender-based analyses where performed, justify reasons for lack of sex- and gender-based analysis.*
Reporting on race, ethnicity, or other socially relevant groupings	*Please specify the socially constructed or socially relevant categorization variable(s) used in your manuscript and explain why they were used. Please note that such variables should not be used as proxies for other socially constructed/relevant variables (for example, race or ethnicity should not be used as a proxy for socioeconomic status). Provide clear definitions of the relevant terms used, how they were provided (by the participants/respondents, the researchers, or third parties), and the method(s) used to classify people into the different categories (e.g. self-report, census or administrative data, social media data, etc.) Please provide details about how you controlled for confounding variables in your analyses.*
Population characteristics	*Describe the covariate-relevant population characteristics of the human research participants (e.g. age, genotypic information, past and current diagnosis and treatment categories). If you filled out the behavioural & social sciences study design questions and have nothing to add here, write "See above."*
Recruitment	*Describe how participants were recruited. Outline any potential self-selection bias or other biases that may be present and how these are likely to impact results.*
Ethics oversight	*Identify the organization(s) that approved the study protocol.*

Note that full information on the approval of the study protocol must also be provided in the manuscript.

Field-specific reporting

Please select the one below that is the best fit for your research. If you are not sure, read the appropriate sections before making your selection.

☒ Life sciences ☐ Behavioural & social sciences ☐ Ecological, evolutionary & environmental sciences

For a reference copy of the document with all sections, see nature.com/documents/nr-reporting-summary-flat.pdf

Life sciences study design

All studies must disclose on these points even when the disclosure is negative.

Sample size	No statistical method was used to predetermine sample size. Live-cell imaging was performed in 2-6 biological replicates, resulting in 12,269-78,268 distances measured. Further statistics are summarized in Supplementary Table 1. Capture Micro-C was performed in two biological replicates, except for the Half TAD and the untagged cell lines where a single replicate was performed, following standard in the field. For FACS, 3 biological replicates were recorded with at least 12,000 cells within the final gate per condition. When bootstrap was used to evaluate the standard deviation of our estimates, we used 5,000-10,000 bootstrap samples. Number of replicates was chosen based on standards in the field.
Data exclusions	No data were excluded from the analysis, except during quality filtering of image time series, as detailed in the 'Quality filtering of trajectories' (see Supplementary Information).
Replication	For live-cell imaging experiments, we performed 2-6 biological replicates with 1-5 technical replicates each. Capture Micro-C was performed

Replication	in two biological replicates, except for the Half TAD and untagged cell lines where a single replicate was performed. For FACS, 3 biological replicates were recorded with at least 12,000 cells within the final gate per condition. Western blot was performed in 3-7 biological replicates.
Randomization	No randomization was performed as the study did not require sample allocation into different groups. Cells imaged in live-cell microscopy were chosen randomly.
Blinding	Blinding was not possible for data collection, as data acquisition required identification of the samples for further processing. Data analysis was not performed blind to the conditions of the experiments, except for Capture Micro-C analysis.

Reporting for specific materials, systems and methods

We require information from authors about some types of materials, experimental systems and methods used in many studies. Here, indicate whether each material, system or method listed is relevant to your study. If you are not sure if a list item applies to your research, read the appropriate section before selecting a response.

Materials & experimental systems

n/a	Involved in the study
☐	☒ Antibodies
☐	☒ Eukaryotic cell lines
☒	☐ Palaeontology and archaeology
☒	☐ Animals and other organisms
☒	☐ Clinical data
☒	☐ Dual use research of concern
☒	☐ Plants

Methods

n/a	Involved in the study
☒	☐ ChIP-seq
☐	☒ Flow cytometry
☒	☐ MRI-based neuroimaging

Antibodies

Antibodies used	Rabbit polyclonal anti-RAD21 Abcam Cat# ab154769, RRID:AB_2783833, Dilution: 1/1500<br>Mouse monoclonal anti-GAPDH Abcam Cat# ab8245, RRID:AB_2107448, Dilution: 1/50000, clone [6C5]<br>Goat anti-rabbit IR800 Advansta R-05060, Dilution: 1/10000<br>Goat anti-mouse IR800 Advansta R-05061, Dilution: 1/10000
Validation	The anti-RAD21 antibody was validated by: (i) a shift in the protein size in cells where the endogenous size was modified with a mAID-SNAP tag, as compared to WT cells,<br>(ii) the absence of detected bands upon auxin-dependent degradation.<br>The GAPDH antibody was tested by the manufacturer (Abcam) on human samples for both Western blot and immunofluorescence.

Eukaryotic cell lines

Policy information about cell lines and Sex and Gender in Research

Cell line source(s)	All cell lines described are derived from WT HCT116 cells from ATCC CCL-247.
Authentication	Cell lines have been recurrently used and have not been authenticated. Homozyguous insertion of repeat arrays showed two fluorescent spots as expected from this diploid cell line.
Mycoplasma contamination	Cells were tested monthly for the presence of Mycoplasma spp., Ureaplasma spp. and A. laidlawii by qPCR.
Commonly misidentified lines (See ICLAC register)	No commonly misidentified cell line was used.

Plants

Seed stocks	*Report on the source of all seed stocks or other plant material used. If applicable, state the seed stock centre and catalogue number. If plant specimens were collected from the field, describe the collection location, date and sampling procedures.*
Novel plant genotypes	*Describe the methods by which all novel plant genotypes were produced. This includes those generated by transgenic approaches, gene editing, chemical/radiation-based mutagenesis and hybridization. For transgenic lines, describe the transformation method, the number of independent lines analyzed and the generation upon which experiments were performed. For gene-edited lines, describe the editor used, the endogenous sequence targeted for editing, the targeting guide RNA sequence (if applicable) and how the editor was applied.*
Authentication	*Describe any authentication procedures for each seed stock used or novel genotype generated. Describe any experiments used to assess the effect of a mutation and, where applicable, how potential secondary effects (e.g. second site T-DNA insertions, mosiacism, off-target gene editing) were examined.*

Flow Cytometry

Plots

Confirm that:

☒ The axis labels state the marker and fluorochrome used (e.g. CD4-FITC).

☒ The axis scales are clearly visible. Include numbers along axes only for bottom left plot of group (a 'group' is an analysis of identical markers).

☒ All plots are contour plots with outliers or pseudocolor plots.

☒ A numerical value for number of cells or percentage (with statistics) is provided.

Methodology

Sample preparation	300,000 cells were seeded and grown in 6-well plates and cells were collected 48 hours later. Cells were trypsinized, washed in PBS, and resuspended in 500 µL PBS. 5.5 mL of ice-cold 70% ethanol was added for fixation.<br>Cells were kept at 4°C in 70% ethanol for at least 12 hours until staining. For staining, cells were washed twice in PBS and incubated for 5 min at room temperature with 50 µL of a 50 µg/mL RNAse A solution (Promega A7973).<br>Finally, 400 µL of 50 µg/mL propidium iodide solution was added and cells were incubated for 15 min at room temperature before FACS sorting.
Instrument	We used a Miltenyi MACSQuant Analyzer 10 Flow Cytometer with the 488 nm laser and a 692/75 nm band pass filter.
Software	FlowJo v10 (BD Biosciences)
Cell population abundance	>12,000 cells in the final gate
Gating strategy	Forward / Side scatters were used to discard large cells with high granularity. Propidium iodide height vs propidium iodide area was used to discard doublets, cells in M phase and cells with a ploidy stricly higher than 2.<br>The histogram of propidium area was then fitted by an unconstrained Dean-Jett-Fox model using FlowJo to obtain the fraction of cells in G1.

☒ Tick this box to confirm that a figure exemplifying the gating strategy is provided in the Supplementary Information.
