# Supplementary material for: Uniform dynamics of cohesin-mediated loop extrusion in living human cells
Source: Nat Genet. 2025 Nov 14;57(12):3152–64. doi: 10.1038/s41588-025-02406-9 (PMC12695666; doi:10.1038/s41588-025-02406-9)
Supplement: Supplementary file 8 — Unprocessed western blots for Extended Data Fig. 3b,d. [file 41588_2025_2406_MOESM8_ESM.pdf]

Source Data - Unprocessed western blot images

Images recorded with different exposure times were used to quantify RAD21 or GAPDH bands

a

Western blots presented in Extended Data Fig. 3b

Exposed to quantify RAD21 | Exposed to quantify GAPDH

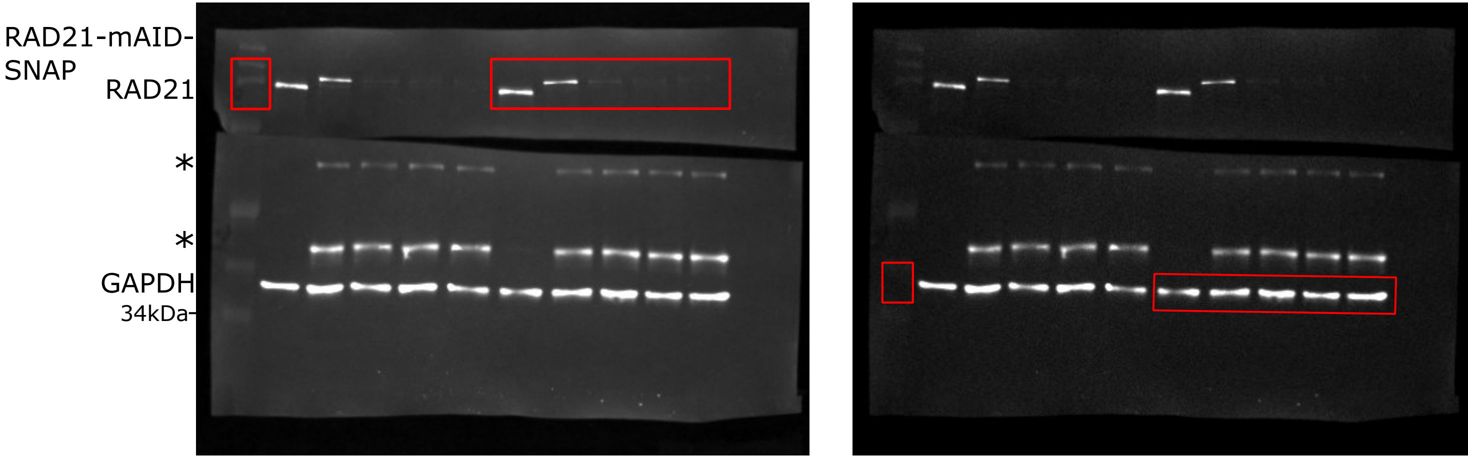

b

Western blots presented in Extended Data Fig. 3d

Exposed to quantify RAD21 | Exposed to quantify GAPDH

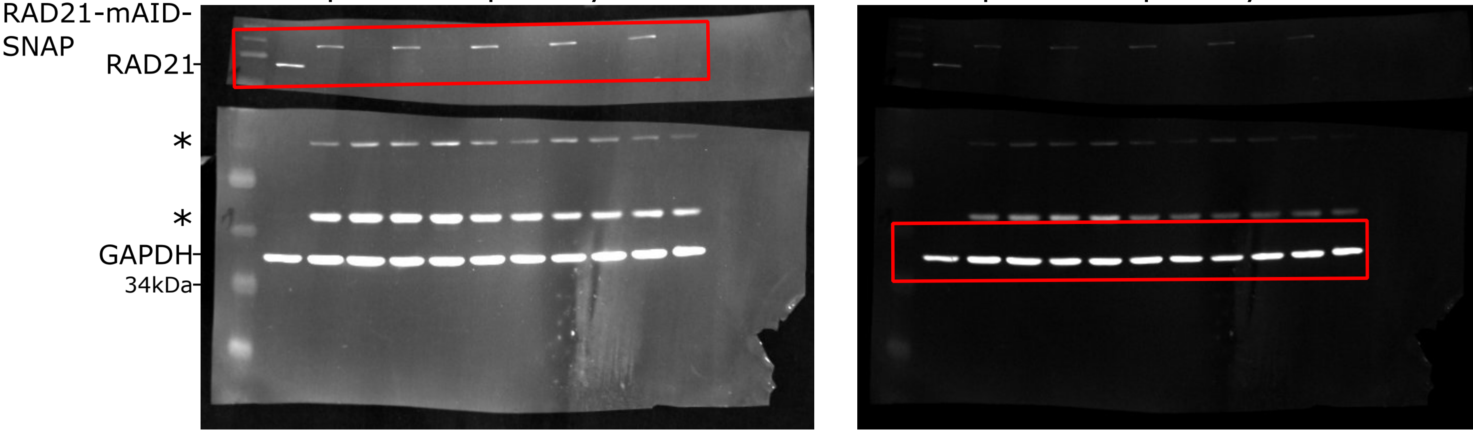

*: GB1 cross-reactivity with secondary antibody as described in Song et al, Frontiers in Plant Science, 2022

Red rectangles show the area represented in the corresponding panels
